# Autosomal dominant cerebellar ataxia type III: a review of the phenotypic and genotypic characteristics

**DOI:** 10.1186/1750-1172-8-14

**Published:** 2013-01-18

**Authors:** Shinsuke Fujioka, Christina Sundal, Zbigniew K Wszolek

**Affiliations:** 1 Department of Neurology at Mayo Clinic, 4500 San Pablo Road Cannaday Bldg 2-E, Jacksonville, FL, 32224, USA; 2 Department of Clinical Neuroscience and Rehabilitation, Institute of Neuroscience and Physiology, Sahlgrenska University Hospital, Gothenburg, Sweden

**Keywords:** SCA5, SCA6, SCA11, SCA26, SCA30, SCA31, SPTBN2, CACNA1A, TTBK2, BEAN

## Abstract

Autosomal Dominant Cerebellar Ataxia (ADCA) Type III is a type of spinocerebellar ataxia (SCA) classically characterized by pure cerebellar ataxia and occasionally by non-cerebellar signs such as pyramidal signs, ophthalmoplegia, and tremor. The onset of symptoms typically occurs in adulthood; however, a minority of patients develop clinical features in adolescence. The incidence of ADCA Type III is unknown. ADCA Type III consists of six subtypes, SCA5, SCA6, SCA11, SCA26, SCA30, and SCA31. The subtype SCA6 is the most common. These subtypes are associated with four causative genes and two loci. The severity of symptoms and age of onset can vary between each SCA subtype and even between families with the same subtype. SCA5 and SCA11 are caused by specific gene mutations such as missense, inframe deletions, and frameshift insertions or deletions. SCA6 is caused by trinucleotide CAG repeat expansions encoding large uninterrupted glutamine tracts. SCA31 is caused by repeat expansions that fall outside of the protein-coding region of the disease gene. Currently, there are no specific gene mutations associated with SCA26 or SCA30, though there is a confirmed locus for each subtype. This disease is mainly diagnosed via genetic testing; however, differential diagnoses include pure cerebellar ataxia and non-cerebellar features in addition to ataxia. Although not fatal, ADCA Type III may cause dysphagia and falls, which reduce the quality of life of the patients and may in turn shorten the lifespan. The therapy for ADCA Type III is supportive and includes occupational and speech modalities. There is no cure for ADCA Type III, but a number of recent studies have highlighted novel therapies, which bring hope for future curative treatments.

## Disease name/synonyms

Autosomal dominant cerebellar ataxias, spinocerebellar ataxias.

## Definition

Autosomal dominant cerebellar ataxias (ADCA) are hereditary neurodegenerative disorders. A number of disease entities present with the ADCA phenotype, such as spinocerebellar ataxias (SCA), dentatorubral-pallidoluysian atrophy, episodic ataxia, and autosomal dominant spastic ataxia. Harding proposed the classification of ADCA into Type I, Type II, and Type III, based on clinical phenotypes in the pre-genetic era [1]. Patients of ADCA Type I exhibit both cerebellar and non-cerebellar signs. ADCA Type I includes SCA1 - SCA4, SCA8, SCA10, SCA12 - SCA23, SCA25, SCA27, SCA28, and SCA32 - SCA36. We have recently reviewed the ADCA Type I in this journal [2]. ADCA Type II comprises syndromes associated with pigmentary maculopathies and includes SCA7. ADCA Type III comprises mostly pure cerebellar syndromes and includes SCA5, SCA6, SCA11, SCA26, SCA30, and SCA31. As an increasing number of cases carrying genes associated with ADCA type III were reported, it has been noted that a small subset of patients of ADCA type III can present with non-cerebellar signs including mild neuropathy, pyramidal signs, or parkinsonism. However, this pre-genetic era classification of ADCA is still important, as it has a prognostic implication. The ADCA Type III patients usually have better prognosis in contrast to ADCA Type I patients. Additionally, this classification allows physicians to more frequently and precisely diagnose patients and save the cost of genetic testing. Table 1 summarizes current status of molecular genetic data for each type of ADCA.

**Table 1:** Genes and genetic loci associated with ADCA types

**Subform**	**Gene**	**Gene product**	**Mutation**	**Locus**
**ADCA Type I**				
SCA1	*ATXN1*	Ataxin 1	CAG repeat	6p22.3
SCA2	*ATXN2*	Ataxin 2	CAG repeat	12q24.12
SCA3	*ATXN3*	Ataxin 3	CAG repeat	14q32.12
SCA4	UN	UN	UN	16q22.1
SCA8	*ATXN8OS*	Ataxin 8	CTG/CAG repeat	13q21.33
SCA10	*ATXN 10*	Ataxin 10	ATTCT repeat	22q13.31
SCA12	*PPP2R2B*	PPP2R2B	CAG repeat	5q32
SCA13	*KCNC3*	KCNC3	Missense	19q13.33
SCA14	*PRKCG*	PRKCG	Missense	19q13.42
SCA15/SCA16	*ITPR1*	ITPR1	Missense, deletion	3p26.1
SCA17	*TBP*	TBP	CAG repeat	6q27
SCA18	UN	UN	UN	7q22-q32
SCA19/SCA22	KCND3	KCND3	Missense, deletion	1p13.2
SCA20	UN	UN	UN	11q12
SCA21	UN	UN	UN	7p21.3-p15.1
SCA23	*PDYN*	prodynorphin	UN	20p13
SCA25	UN	UN	UN	2p21-p13
SCA27	*FGF14*	FGF14	Missense, frameshift	13q33.1
SCA28	*ATG3L2*	ATG3L2	Missense	18p11.21
SCA32	UN	UN	UN	7q32-q33
SCA34	UN	UN	UN	6p12.3-q16.2
SCA35	*TGM6*	TGM6	Missense	20p13
SCA36	*NOP56*	NOP56	GGCCTG repeat	20p13
**ADCA Type II**				
SCA7	*ATXN7*	Ataxin 7	CAG repeat	3p14.1
**ADCA Type III**				
SCA5	*SPTBN2*	SPTBN2	Inframe deletion, missense	11q13.2
SCA6	*CACNA1A*	CACNA1A	CAG repeat	19q13.2
SCA11	*TTBK2*	TTBK2	Stop, frameshift insertion, frameshift deletion	15q15.2
SCA26	UN	UN	UN	19p13.3
SCA30	UN	UN	UN	4q34.3-q35.1
SCA31	*BEAN-TK2*	BEAN	TGGAA repeat	16q21

ADCA: autosomal dominant cerebellar ataxia; AFG3L2: ATPase family gene 3-like 2; ATSN8OS: ataxin 9 opposite strand; ATN: atrophin; ATXN: ataxin; BEAN: brain expressed, associated with Nedd4; CACNA1A: calcium channel, voltage-dependent, P/Q type, alpha 1A subunit; CBMC: cord blood-derived mononuclear cells; EAAT4: excitatory amino acid transporter; FGF14: fibroblast growth factor 14; ITPR1: inositol 1,4,5-triphosphate receptor 1; KCNC3: potassium voltage-gated channel subfamily C member 3; KCND3: potassium voltage-gated channel, shal-related subfamily, member 3; MRI: magnetic resonance imaging; NOP56: NOP56 ribonucleoprotein homolog; PPP2R2B: protein phosphatase 2, regulatory subunit B, beta isoform; PRKCG: protein kinase Cγ; RNAi: RNA interference; SCA: spinocerebellar ataxia; siRNA: small interfering RNA; SPTBN2: spectrin, beta, non-erythrocytic 2; TATA: thymine adenosine thymine adenosine; TBP: TATA box binding protein; TGM6: transglutaminase 6; TK2: thymidine kinase 2; TTBK2: tau tubulin kinase-2; UN: unknown.

This review focuses on ADCA Type III. ADCA Type III currently is comprised of a group of six disorders.

## Clinical description

ADCA Type III, as a group, is a relatively benign and slowly progressing set of disorders. It is clinically characterized by mostly pure cerebellar signs including gait, stance, and limb ataxia as well as dysarthria. Affected subjects present with cerebellar oculomotor dysfunction, such as nystagmus and impaired smooth pursuit. The characteristics of oculomotor dysfunctions may vary between each subtype. Non-cerebellar signs including pyramidal features, peripheral neuropathy, involuntary movements, and others, are occasionally seen in ADCA Type III. We discuss the clinical phenotype of each subtype below.

## Epidemiology

The prevalence of ADCA Type III is unknown; however, studies have estimated that the incidence may be variable based upon geographical location/population. There were estimated to be three ADCA cases per 100,000 people in the Netherlands [3], and 4.2 ADCA cases per 100,000 people in Norway [4].

SCA6 is the most common subtype of ADCA Type III. Additionally, SCA6 is one of the most common subtypes of all three types of ADCA, accounting for approximately 13% of all cases[5]. The prevalence of SCA6 is estimated to be less than one per 100,000. SCA6 is commonly seen in Japan (6-32%) [6-11], Korea (15-23%) [12,13], the Netherlands (11-23%) [14,15], Germany (10-22%) [16-18], and less commonly observed in UK (5%) [19], India (0-4%) [20,21], China (0-3%) [22,23], South Africa (2%) [24], Thailand (2%) [25], Italy (1-2%) [26,27], France (1%) [28], Finland (1%) [29], and Spain (1%) [30]. No cases of SCA6 have been reported in Portugal [31,32]. SCA31 is the second most common ADCA subtype and is mainly seen in Japan (9%) [33]. The prevalence of SCA5, SCA11, SCA26, and SCA30 are reported to be relatively rare. Founder effects have likely contributed to the variable prevalence between populations. The variation in number of normal triplet repeats in populations, as also observed in other triplet repeat disorders such as Huntington disease, may influence the variability of prevalence between populations. Epidemiological features of ADCA Type III are summarized in Table 2.

**Table 2:** Epidemiological findings of ADCA Type III

**Subform**	**Country**	**Reported frequency of each ADCA subform**			
		**Common (>20%)**	**Relatively common (5-20%)**	**Rare (0<, <5%)**	**None (0%)**
SCA5	USA, German, France	-	-	USA, German, France	-
SCA6	Many	Japan, Netherland, Korea, German	USA, Taiwan, Australia	UK, India, China, Thailand, Italy, France, Finland, Spain, South Africa	Portugal
SCA11	England, German, France	-	-	France, German	China
SCA26	USA	-	-	-	-
SCA30	Australia	-	-	-	-
SCA31	Japan	-	Japan	Korea	USA, China

ADCA: autosomal dominant cerebellar ataxia; SCA: spinocerebellar ataxia; UK: the United Kingdom; USA: the United States of America.

## Molecular genetics and etiology

The pathogenesis of the ADCA Type III is not fully understood. There are currently four causative genes, which have been identified to have an association with ADCA Type III. Conventional mutations in the *spectrin, beta, non-erythrocytic 2* (*SPTBN2*) gene for SCA5, polyglutamine expansion in the *calcium channel, voltage-dependent, P/Q type, alpha 1A subunit* (*CACNA1A*) gene for SCA6, conventional mutations in the *tau tubulin kinase-2* (*TTBK2*) gene for SCA11, and non-coding expansions in the *brain expressed, associated with NEDD4* (*BEAN*) gene for SCA31. These can all cause ADCA Type III. In addition, two loci have been discovered for the other subforms, SCA26 and SCA30. We will discuss the molecular genetics and etiology of each subform later in the text.

## Diagnosis and differential diagnosis

**Figure 1:**
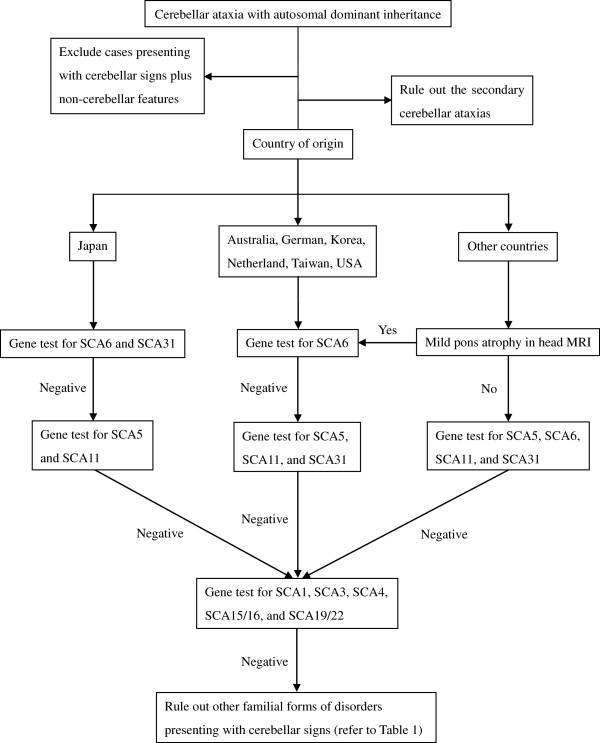
**This flowchart explains diagnostic algorithm for ADCA Type III.** After excluding the cases presenting with cerebellar signs plus non-cerebellar features and secondary cerebellar ataxias, we suggest the appropriate genetic testing based on geographic distribution (patient’s country of origin). If the genetic tests are negative for known ADCA Type III gene mutations, we suggest to perform genetic testing for subtypes of ADCA Type I, which could possibly present with pure cerebellar syndrome at the early stage of illness. After excluding these diseases, other familial forms of disorders presenting with cerebellar signs should be considered. ADCA: autosomal dominant cerebellar ataxia; MRI: magnetic resonance imaging; SCA: spinocerebellar ataxia; USA: the United States of America.

There are no fully validated diagnostic criteria for ADCA Type III or for any of the other forms of pure cerebellar ataxias. A definitive diagnosis is based on genetic testing; however, a clinical history, family history of similar phenotype, physical examination, and neuroimaging, including head magnetic resonance imaging (MRI) are all important in obtaining the precise diagnosis. When seeing ataxic patients, it is crucial to discern whether they have pure cerebellar ataxia or cerebellar ataxia plus additional non-cerebellar symptoms. If patients manifest pure cerebellar ataxia, then the next step is to exclude any secondary causes such as drug side effects, toxicity, nutritional deficits, infections, and structural abnormalities. It is noted that cerebellar atrophy on MRI is an important argument in favor of a neurodegenerative disease diagnosis such as ADCA in contrast to a secondary cause of cerebellar ataxia. Secondary cerebellar ataxias have been previously discussed [2]. After alternate causes for ataxia have been excluded, genetic testing may be conducted for a definitive diagnosis. In Japan, SCA6 is the most common subtype of ADCA type III, followed by SCA31. Therefore, genetic testing for these genes should be considered first. In Australia, Germany, Korea, the Netherlands, Taiwan, and the USA, SCA6 is the most common or is relatively common. Therefore, genetic testing for SCA6 should be considered first. In other countries, such as China, Finland, and France, ADCA Type III is a rare disorder or the prevalence of ADCA Type III is unknown. It is recommended that a head MRI be performed. If mild pons atrophy exists, genetic testing for SCA6 could be considered first. If there is no pons atrophy, then genetic tests for SCA5, SCA6, SCA11, and SCA31 are recommended. If the genetic tests are negative for known ADCA Type III, we suggest to perform genetic testing for SCA1, SCA3, SCA4, SCA15/16 and SCA19/22. These subtypes could present with pure cerebellar syndrome at the early stage of illness. After excluding the mutations in these genes, other familial forms of disorders presenting with cerebellar signs should be ruled out. The strategy for diagnosis of cerebellar ataxia is summarized in Figure 1.

## Management including treatment

There is currently no cure for ADCA Type III or its subtypes. Supportive care still remains the mainstay of management; however, a variety of different kinds of treatments are emerging.

## Supportive therapies

It is important for patients with ADCA Type III to be involved in physical and occupational therapies from the onset of their gait dysfunction or dysarthria. Computer devices are useful for communication in subjects with severe dysarthria. Mechanical aids such as a cane, walker, or wheelchair can allow the patient to remain both mobile and safe.

## Pharmacological therapies

Several clinical trials have been conducted for potential SCA6 therapies. Yabe and colleagues showed that acetazolamide (250-500 mg/day) temporally, but significantly, reduced the severity of ataxia in SCA6 patients [34]. A pilot trial revealed that gabapentin (1200 mg/day) alleviated some of the ataxia symptoms in SCA6 [35]. An open-label trial with tandospirone (15 mg/day) for SCA6 patients showed a reduction in the total score on the ataxia rating scale and total length traveled by SCA6 patients [36]. In this study, the length travelled was defined as the movement in distance per minute (m/s) of the patient’s center of gravity as calculated by the software incorporated in the stabilometer (Gravicorder, Model G5500; Anima Corp, Tokyo, Japan).

## Gene therapy

RNA interference (RNAi) aimed at post-transcriptional silencing of disease causing a selective degradation of mRNA, has attracted interest as a new emerging therapeutic option [37]. This novel therapy has already been applied to some neurodegenerative conditions including polyglutamine diseases. Xia and colleagues described that intracerebellar injection of RNAi successfully led to improvement of motor coordination in the mouse models of SCA1 [38]. They also found that it restored cerebellar morphology and resolved characteristic inclusions in the Purkinje cells of the mouse model. Scholefield and colleagues showed that selective silencing of mutant Ataxin 7 caused significant reduction in the levels of the toxic mutant Ataxin 7 in cells [39]. Recently, pioneering work has been conducted that may lead to the development of potential RNAi therapies for one of the subtypes, SCA6. Tsou and colleagues developed a novel splice isoform-specific-RNAi strategy that selectively targets the polyQ-encoding Cav2.1 splice valiant [40]. They achieved the selective suppression of the polyQ-encoding Cav2.1 splice variants utilizing a new artificial microRNA-like delivery system. So far, an increasing number of reports, including these studies, have been published associated with RNAi therapies. However, numerous problems such as selection of potent siRNAs, the safety and efficacy of these compounds, and the eventually drug delivery to tissues or cells, remain to be elucidated before they can become clinically available [41].

## Stem cell therapy

An increasing number of studies have proven the efficacy of stem cell therapy for animal models of a variety of diseases, including cerebellar ataxia [42-44]; however, stem cell therapy still has not been shown to be a proven therapy in humans and is not recommended at this time.

## Exercise

The beneficial effects of routine exercise have been reported not only for metabolic diseases, but also neurodegenerative disorders such as Alzheimer’s disease [45,46] and Parkinson’s disease [47]. Fryer and colleagues have shown that exercise can improve motor impairment, as well as learning and memory deficits in the ATXN-1 mouse model [48]. Ilg and colleagues reported that intensive coordinative training significantly improved motor performance and alleviated symptoms in patients with cerebellar degeneration, including SCA6 subjects [49,50]. However, it is still unclear whether excise has an influence on the origin of the disease directly or if it is simply supportive care.

## Others

Subcutaneous insulin-like growth factor-1 treatment [51] and transglutaminase inhibitor [52,53] treatment for ataxic disorders are being evaluated in pre-clinical and clinical trials. However, they are not yet available for ADCA Type III patients.

## Prognosis

As a group ADCA Type III, progresses slowly and are not life-threatening. However, there is possible intra or inter familial variability. Having dysphagia or frequent falls may shorten the lifespan of the patient.

## Clinical description, Molecular genetics, and etiology of each subform

### Spinocerebellar Ataxia Type 5 (SCA5)

Three families, American, German, and French have been reported [54-56]. SCA5 has age related penetrance. The age of symptomatic disease onset is between 10 and 68 years (mean 33 years) without anticipation [54]. This slowly progressive type of ADCA can have a disease duration of more than 30 years. SCA5 presents with cerebellar signs and eye movement abnormalities, including down beat nystagmus, gaze-evoked nystagmus, and impaired smooth pursuit. Several patients manifested non-cerebellar signs such as facial myokimia, horizontal gaze palsy, intention or resting tremor, brisk deep tendon reflexes, and impaired vibration sense [56,57]. Head MRI shows global atrophy of the cerebellum without any involvement of brainstem or any other brain regions [57].

In 1994, Ranum and colleagues mapped the locus on chromosome 11 by linkage analysis in a large American family affected by dominant ataxia [54]. In 2004, Bürk and colleagues narrowed the *SCA5* locus to a 5.15-Mb interval on chromosome 11q13 [56]. In 2006, Ikeda and colleagues discovered the *SPTBN2* mutations, encoding β-III spectrin, in the original American kindred and two additional kindreds [58]. To date, two in-frame deletions and one missense mutation have been confirmed as pathogenic mutations [58]. β-III spectrin consists of 2390 amino acid proteins and is predominantly expressed in Purkinje cells [59,60] and stabilizes the glutamate transporter, excitatory amino acid transporter (EAAT4), at the plasma membrane [58]. *SPTBN2* mutations were found to cause impaired axonal transport in Drosophila [61]. In addition, the loss of β-III spectrin reduced the spontaneous firing rate in surviving Purkinje cells and deregulated the glutamatergic neurotransmission in mice [62]. Clarkson and colleagues found that a β-III spectrin L253P mutation interferes with binding to Arp1, a subunit of the dynactin-dynein complex, and disrupts protein trafficking of both β-III spectrin and EAAT4 from the Golgi [63]. However, the precise mechanism of β-III spectrin function has yet to be elucidated.

## Spinocerebellar Ataxia Type 6 (SCA6)

SCA6 is the most common subtype in ADCA type III and the second most common subtype in all types of ADCA, including ADCA Type I, ADCA Type II, and ADCA Type III. Incidence of SCA6 varies in the worldwide population. SCA6 is a late-onset and slowly progressive form of ataxia [6]. Some affected individuals can walk without any assistance more than 20 years after disease onset [55,64,65]. A prospective natural history study using affected SCA6 patients as well as patients from the three other subtypes of ADCA Type I, including SCA1, SCA2, and SCA3 conducted by European Integrated Project on Spinocerebellar Ataxias revealed that the disease progression was slowest in SCA6 [66]: increased SARA score [67] was 0.35±0.3 for one year. The age of symptomatic disease onset is between 16 and 72 years (mean age: 45 years). Approximately 60% of patients develop disease after age 50 years [5]. Penetrance is almost 100% [68]. Anticipation has not been observed [69]. Disease duration can be more than 25 years. SCA6 is mainly characterized by cerebellar signs as well as eye movement problems, such as gaze-evoked nystagmus, downbeat nystagmus, impaired vestiblo-ocular reflex, and impaired smooth pursuit. The majority of SCA6 patients develop gait ataxia as an initial symptom. Some patients manifest episodic vertigo, diplopia, and dysarthria prior to gait abnormalities [70]. SCA6 occasionally presents with extracerebellar symptoms, such as pyramidal tract signs [17] and peripheral neuropathy [3]. Occasionally, cognitive impairment [71], parkinsonism characterized by bradykinesia [72], myoclonus, dystonia, tremor including postural, action, and terminal tremor of heads, or other movement disorders may be seen [73]. In addition, depression [74] and fatigue [75] may be associated with SCA6. Head MRI reveals severe cerebellar atrophy accompanied by mild atrophy of the middle cerebellar peduncle, pons, and red nucleus [76,77]. Single-photon emission computed tomography using *N*-isopropyl-*p*^123^I]iodoamphetamine shows decreased tracer uptake in the cerebellum [78]. Positron emission tomography studies with ^18^F]Fluorodeoxyglucose reveal that the glucose metabolism rate was reduced not only in cerebellum and brainstem, but also in cortical regions and basal ganglia [79].

In 1997, Zhuchenko and colleagues identified small expansions of the trinucleotide (CAG)_n_ repeat in the *CACNA1A* gene on chromosome 19p13, that encoded the α1 subunit of a P/Q-type voltage-gated calcium channel. Expanded alleles usually have 20 to 29 CAG repeats [8,69,80], whereas the normal alleles have 4 to 18 repeats [80]. Mariotti and colleagues described that affected subjects who were homozygous for an intermediate allele of 19 CAG repeats in the *CACNA1A* gene [81]. CACNA1A exists in granule cells and Purkinje cells of the cerebellar cortex. The central role of CACNA1A is thought to be in synaptic transmission. It is assumed that polyglutamine repeats in CACNA1A effects Ca^2+^ channel to reduce Ca^2+^ influx, leading to eventually cell death [82]. The polyglutamine repeats in SCA6 are much smaller than in others harboring polyglutamine expansion SCAs [83]. It has yet to be determined whether such small expansions can cause pathological effects in normal CACNA1A function by altering the calcium channel function or if it acquires a new toxic function [84,85].

## Spinocerebellar Ataxia Type 11 (SCA11)

SCA11 is another rare subtype of ataxia. To date, four families, a British family from Devon, UK, a British family of Pakistani ancestry, a German family, and a French family, have been reported [86-88]. In two additional studies, SCA11 was not observed in 68 unrelated Han Chinese patients [89] or 48 unrelated familial cases of German descent [90]. SCA11 presents with early-onset, and slowly progressing cerebellar symptoms. The age of symptomatic disease onset is between 11 and 70 years (mean: 25 years). This disease may have full penetrance [87]. Disease duration is over 20 years and some cases remain ambulant for up to 16 years after onset [88]. SCA11 is clinically characterized by cerebellar signs and eye movement abnormalities, which include jerky pursuit, ophthalmoplegia, and horizontal and vertical nystagmus. Occasionally, affected patients manifest mild to moderate hyperreflexia, especially in lower limbs but with negative Babinski signs [87]. Peripheral neuropathy and dystonia may also be seen [86]. Head MRI shows isolated marked cerebellar atrophy [86,87].

In 1999, Worth and colleague mapped the locus on chromosome 15q14-21 in two British families with the ADCA phenotype [86]. In 2007, Houlden and colleagues identified two *TTBK2* mutations, one is a 1-base insertion of an adenosine in exon 13 at nucleotide 1329, codon 44; another is a frameshift deletion of a 2-base guanine and adenosine in exon 13. TTBK2 mRNA is expressed in all brain regions, especially in Purkinje cells, granular cell layer, hippocampus, midbrain, and the substantia nigra [87]. TTBK2 phosphorylates the tau protein and stabilizes Purkinje cells [87]. Mutant TTBK2 interrupts normal phosphorylation of tau protein and eventually causes tau deposition, particularly in Purkinje cells.

## Spinocerebellar Ataxia Type 26 (SCA26)

SCA26 is very rare subtype. Only one American family of Norwegian descent has been reported. This family has 23 affected family members and 14 at-risk members [91]. The age of symptomatic disease onset is between 26 and 60 years (mean of 42 years) without anticipation. Disease duration is still unknown. SCA26 presents with relatively late-onset, slowly progressive cerebellar symptoms and eye movement abnormalities. Eye movement abnormalities are characterized by impaired pursuit and nystagmus. Only one patient presented with left-sided hyperreflexia with positive Babinski sign. Head MRI showed isolated cerebellar atrophy.

In 2005, Yu and colleagues mapped a 15.55-cM locus on chromosome 19p.33.3 by a genome-wide linkage analysis of a large American family with the ADCA phenotype [91]. This locus is closed to *CACNA1A*, the causative gene for SCA6; however, *CACNA1A* is also about 19-cM centromeric to locus of SCA26. The responsible gene for SCA26 is still unknown.

## Spinocerebellar Ataxia Type 30 (SCA30)

SCA30 is very rare subtype and only one Australian family with six affected subjects has been reported to date [92]. The age of symptomatic disease onset is between 45 and 76 years (mean 52 years). SCA30 is clinically characterized by relatively pure and slowly progressive cerebellar ataxia. Several affected subjects had mild hyperreflexia in their lower limbs. One case presented with gaze-evoked nystagmus. Another affected patient also had dystonia. Of note, several deceased family members may have had parkinsonism according to family histories; although, the details of their clinical features were unavailable. Head MRI showed isolated atrophy of cerebellum, predominantly superior and dorsal cerebellar vermis.

In 2009, Storey and colleagues mapped a 5-Mb locus on chromosome 4q34.3-q35.1 by a genome-wide linkage analysis of an Australian family with the ADCA phenotype [92]. The causative genetic mutation has yet to be discovered.

## Spinocerebellar Ataxia Type 31 (SCA31)

SCA31 is rare subtype of ADCA type III except in Japan, where it is the fourth most common form of ADCA[33,93,94]. More than 20 families have been reported from Japan to date [93,95,96]. SCA31 presents with a late-onset progressive form of ataxia. The age of symptomatic disease onset is between 8 and 83 years with mean of about 58 years. The disease duration is more than 10 years [94]. The phenomenon of anticipation is absent or probably mild [95]. This disease is believed to have incomplete penetrance. [93]. SCA31 is clinically characterized by cerebral ataxia and eye movement abnormalities, such as horizontal gaze nystagmus and impaired pursuit. Occasionally, affected subjects manifest pyramidal signs [93], hearing difficulties [93,96], and decreased vibration [93]. Occasionally, tremor [94] may be seen. Head MRI showed global atrophy of the cerebellum, but in a few cases cerebral atrophy was also present [94].

In 2000, Nagaoka and colleagues mapped a locus to chromosome 16q [95] by a genome-wide linkage analysis of six Japanese families [95]. In 2004, Hirano and colleagues refined the candidate locus to a 1.25-Mb interval on chromosome 16q22.1 [97]. This locus is also the candidate interval of SCA4, though the clinical phenotypes differ from each other. Ishikawa and colleagues identified a single-nucleotide change in the *PLEKHG4* gene in 109 affected patients and in 48 at-risk individuals from 52 families [98]; however, other studies failed to detect this change in 16p22.1-linked ADCA patients [99,100]. In 2009, Sato and colleagues discovered 2.5 to 3.8 kb insertions of penta-nucleotide repeats, (TGGAA)n, (TACAA)n, and (TAAAA)n, on chromosome 16q21-q22 using southern blot analysis and sequencing analysis in 160 affected individuals from 98 families [101]. Among these repeats, (TGGAA)n is thought to be pathogenic in Japanese subjects. In the study of the European population, all expansions had pure stretches of (TACAA)n, (GAAAA)n or (TACAA)n in their expanded alleles, without any expansion identified in Japanese series [102]. This repeat exists in an intronic region shared by two genes, *BEAN* and *TK2*. This insertion was not observed in control subjects or in individuals with SCA4. The length of the insertion is inversely correlated with the age at symptomatic disease onset; therefore, the length of inserted TGGAA repeat seems to be associated with the toxicity.

Clinical features of ADCA Type III are summarized in Table 3.

**Table 3:** Clinical features of ADCA Type III

**Subform**	**N. of Pt**	**References**	**AAO (range)**	**Clinical phenotype**			**Atrophy**
				**Common (>50%)**	**Occasional (10<, <50%)**	**Rare (<10%)**	
SCA6	465	[8,10,15,17,22,55,64,65,68,69,78,103-111]	45 (16–72)	A, D, nystagmus^#^	GEN	IVOR, ISP, ophthalmoplegia, SS, PTS, CI, myoclonus, dystonia, tremor, rigidity, EA	Pancerebellar, pons, cerebellar peduncle, red nucleus
**Subform**	**N. of Pt**	**References**	**AAO (range)**	**Clinical phenotype**			**Atrophy**
				**Common (>50%)**		**Uncommon (<50%)**	
SCA5	31	[54,56,57]	33 (10–68)	A, D, IVOR, GEN		DBN, hyperreflexia, resting tremor, intension tremor, facial myokimia, ophthalmoplegia, tremor, DVS	Pancerebellar
SCA11	21	[86-88]	25 (11–70)	A, D, ISP, nystagmus, hyperreflexia		ISP, DVS, GEN, IVOR	Pancerebellar
SCA26	15	[91]	42 (26–60)	A, D, ISP		Nystagmus, hyporeflexia	Pancerebellar
SCA30	6	[92]	52 (45–76)	A, D, hyperreflexia		GEN, dystonia	Pancerebellar*
SCA31	114	[33,93,95-97]	58(8–83)	A,D, nystagmus, GEN		DVS, Hyperreflexia, spasticity, hearing difficulty, hyporeflexia, tremor	Pancerebellar**

A: ataxia; AAO: age at onset; ADCA: autosomal dominant ataxia; CI: cognitive impairment; D: dysarthria; DVS: decreased vibration sense; EA: episodic ataxia; GEN: gaze evoked nystagmus; ISP: impaired smooth pursuit; IVOR: impaired vestibulo-ocular reflex; N: number; PN: peripheral neuropathy; Pt: patients; PTS: pyramidal tract signs; SCA: spinocerebellar ataxia; SS: slow saccades; *: predominant in superior and dosal cerebellar vermis; ^**^: a few cases showed cerebral atrophy; ^#^: down beat nystagmus is frequent.

## Conclusions

In our review, we describe the clinical, genetic, molecular, and phenotypic aspects of ADCA Type III. There has been remarkable progress in the understanding of the genetic and molecular mechanisms associated with ADCA. Additionally, genetic testing for this disease is becoming less costly and more widely available due to the technological advancements of genetic sequencing. However, the Harding classification is still very important, because collecting the essential clinical phenotype and selecting the most appropriate genetic tests are crucial for the diagnosis of cerebellar ataxias. To remain cost effective, this requires efficient clinical disease classification, such as Harding’s, and well-organized diagnostic criteria that narrow the diagnostic possibilities.

ADCA Type III is a rare group of neurodegenerative disorders with the exception of SCA6. The clinical phenotype, pathological characteristics, and biomarkers associated with ADCA Type III are still not well understood. Moving forward, the greatest challenges for future research are the identification of families with ADCA Type III phenotype without known mutations, identification of causative genes and pathogenesis, and the development of specific treatments. Hopefully, such efforts will eventually lead to the identification of curative treatments for ADCA Type III.

## Abbreviations

ADCA: Autosomal Dominant Cerebellar Ataxia; BEAN: Brain Expressed, Associated With Nedd4; CACNA1A: Calcium Channel, Voltage-Dependent, P/Q Type, Alpha 1A Subunit; EAAT4: Excitatory Amino Acid Transporter; MRI: Magnetic Resonance Imaging; PLEKHG4: Pleckstrin Homology Domain-Containing Protein, Family G, Member 4; RNAi: RNA interference; SCA: Spinocerebellar Ataxia; siRNA: small interfering RNA; SPTBN2: Spectrin, Beta, Non-Erythrocytic 2; UK: United Kingdom; USA: United States of America; TK2: Thymidine Kinase 2; TTBK2: Tau Tubulin Kinase-2.

## Competing interests

The authors declare that they have no competing interests.

## Authors’ contributions

SF wrote the first draft of this paper; CS and ZKW reviewed and revised the manuscript for intellectual content. All authors read and approved the final manuscripts.

## Authors' information

SF is a research fellow in the Department of Neurology at the Mayo Clinic in Jacksonville, Florida. He received a medical degree from Fukuoka University, Fukuoka, Japan. He completed his internship at Fukuoka University and moved to Tokyo where he completed his neurology residency training at Tokyo Rosai Hospital, Tokyo, Japan. After passing the Japanese Neurology Board Certification, SF moved to Mayo Clinic Florida in 2010, where he started his clinical research. His research interests include neurodegenerative disorders, clinical genetics and pathology. SF has received fellowship to attend the Aspen Course of Movement Disorder in 2011. SF is a member of American Academy of Neurology, Japanese Society of Neurology, the Japanese Society of Internal Medicine and the Japanese Stroke Society.

CS is a research fellow from the Institute of Neuroscience and Physiology, Department of Clinical Neuroscience and Rehabilitation, Sahlgrenska Academy, University of Gothenburg, Gothenburg, Sweden. She received a medical degree from University of Bergen, Norway. She completed her neurology residency training at Sahlgrenska University Hospital, Gothenburg, Sweden. CS completed a one year rotationtraining as a neurology fellow at Mayo Clinic in Jacksonville, Florida in 2011. Her research interests focus on neurodegenerative disorders,brain white matter disorders clinical genetics and biomarkers. CS has received fellowship to attend the Aspen Course of Movement Disorders in 2011 and a travel award for the annual meeting of American Neurological Association in 2011. CS is a member of American Academy of Neurology, the Swedish Neurological Society, the Swedish Medical Association, the Norwegian Medical Association, and the Swedish Epilepsy Society.

ZKW is a professor and consultant in the Department of Neurology at the Mayo Clinic in Jacksonville, Florida. He received a medical degree from Silesian Medical University, Katowice, Poland. ZKW also finished an internship in Internal Medicine and completed his residency in Neurology at the University of Nebraska Medical Center, Omaha, NE, USA. He is a neurologist with more than 30 years of clinical experience. His research interests focus on neurodegenerative disorders as well as, clinical genetics, biomarkers, and therapeutic approaches. Based on the kindred studies that ZKW has conducted for more than 25 years, several important genetic discoveries have been made. These include the discovery of *MAPT*, *LRRK2*, *DCTN1, VPS35*, *EIF4G1*, *C9ORF72*, *CSF1R and CIZ1* genes, among others. ZKW has directed a the clinical core for the NIH/NINDS Morris K. Udall Center of Excellence for PD Research grant awarded to the Mayo Clinic Florida since 1999 to present. ZKW has received Annemarie Opprecht-Foundation award from Switzerland in 2005 Parkinson Society Canada’s Donald Calne Lecturship Award, an honorary membership of the Polish Neurological Society, the 2009 Distinguished Mayo Investigator Award for the Mayo Clinic Florida, the John A. Beals Awardof the Duval County Medical Society, and the Fifth Annual Robert W. Hervey Distinguished Lecture on Parkinson’s Disease Award: Methodist Neurological Institute of,Houston, Texas. ZKW is a member of American Neurological Association, American Academy of Neurology, International association of Parkinsonism and Related Disorders, the Movement Disorder Society, American Clinical Neurophysiology Society, American Association of Electrodiagnostic Medicine, Mayo Alumni Association, Parkinson Study Group, and Duval County Medical Society. ZKW has served as an editor-in-chief or associated editor on three neurological journals, *Parkinsonism and Related Disorders*, *European Journal of Neurology*, and Polish edition of *Neurology*.
